# Leadless Pacemaker: a New Concept in Cardiac Pacing

**DOI:** 10.5935/abc.20160144

**Published:** 2016-10

**Authors:** Nicodemus Lopes, Diogo Cavaco, Pedro Carmo, Maurício Ibrahim Scanavacca, Pedro Adragão

**Affiliations:** 1 Centro Hospitalar de Lisboa Ocidental - Lisboa - Portugal; 2 Instituto do Coração (InCor), HCFMUSP, São Paulo - Brazil

**Keywords:** Atrioventricular Block, Cardiac Pacing, Artificial, Pacemaker, Artificial, Catheterization, Central Venous

67 year-old female patient, pacemaker user since 1983 due to complete atrioventricular block. Between 1991 and 2004, the patient underwent six surgeries. The first was to replace the batteries, and the others for electrode displacement, generator extrusion, and finally for endocarditis, when surgical extraction of the system and epicardial electrode implantation in the right ventricle (RV) were performed. In the last assessment, in 2015, the patient presented with a very high ventricular stimulation threshold and battery depletion. Due to previous technical problems, implantation of transvenous leadless pacemaker was considered (Micra-Medtronic). This new cardiac stimulation system has, as its main characteristics, reduced generator size (volume of 0.8 cm^3^) and the absence of electrodes, making it possible to perform the implantation of the system directly in the RV. Implantation was performed at Hospital Santa Cruz, Carnaxide, Portugal. After local anesthesia, a sheath (23F) was introduced through the right femoral vein to take the system to the RV. Once inside the ventricle, the delivery catheter was directed to the septal and apical portion and the capsule was released. Fixation of the system in the right ventricle trabeculae, through its flexible tines, was confirmed by mechanical fixation tests. After that, electronic assessment was performed yielding good parameters. After ensuring that parameters were adequate and the capsule was well fixated, the capsule was released and the delivery system was removed. The patient did not present any immediate complications, and after 45 days of follow-up, electronic parameters were stable.

**Figure 1:**
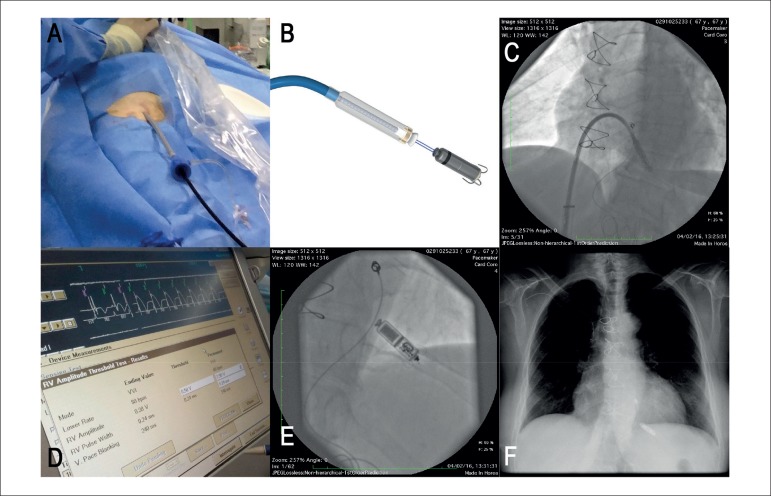
A) System used to implant the leadless pacemaker in the right ventricle. B) Micra at the tip of the catheter. C) Positioning of Micra in the left oblique view: apicalseptal region. D) Assessment of intraoperative parameters. E) Final position of the leadless pacemaker. F) Chest X-Ray on the first day after implantation.

